# An Energy-Efficient Edge Vision System for Front-End Quality Monitoring in Sustainable Cold Chains: Coffee Fruit Ripeness Detection and Embedded Validation

**DOI:** 10.3390/s26206535

**Published:** 2026-10-15

**Authors:** Xi Cui, Shirong Guo

**Affiliations:** 1 Faculty of Engineering, Monash University, Clayton, VIC 3800, Australia; xcui0033@student.monash.edu; 2 Faculty of Business and Economics, Monash University, Melbourne, VIC 3145, Australia

**Keywords:** sustainable cold chains, visual quality sensing, edge artificial intelligence, neural processing units, coffee fruit ripeness

## Abstract

Reliable quality monitoring at the front end of sustainable cold chains requires direct observations of product condition in addition to environmental measurements. This study developed an energy-efficient edge vision system for six coffee fruit ripeness and visible-condition classes on an RK3576 neural processing unit (NPU). YOLO11 was reconfigured by combining a Branch-Integrated Feature Aggregation module for fine-grained representation, a YOLOv10-derived Cross-Stage Partial Network with Compact Inverted Block for efficient multi-scale fusion, and a Simplified Regression Head with reduced regression channels. On a public benchmark derived from 996 source images, YOLO11 and YOLO11BCS yielded mean average precision values at an intersection-over-union threshold of 0.5 (mAP50) of 81.5% and 83.2%, respectively, under the matched 320-pixel protocol. At a 640-pixel input, the computational complexity decreased from 6.3 to 5.5 giga floating-point operations. The largest class-level mAP50 difference was 9.7 percentage points for overripe fruit. Under controlled synthetic degradations, the model produced higher point estimates than YOLO11 for illumination variation and rain, whereas severe Gaussian noise and motion blur remained limiting. Board-input measurements showed throughput increases of 24.2% and 22.1% at 640- and 320-pixel inputs, respectively. The 320-pixel system reached 50.3 frames per second and 10.06 frames per second per watt. Within the supplied benchmark, the matched evaluation supports a comparative assessment of the models, while source-image correspondences across partitions define the scope of the reported absolute metrics. Within these boundaries, the results support low-power visual quality monitoring near the point of harvest.

## 1. Introduction

Perishable-food quality changes from harvest through grading, precooling, packaging, transport and storage, so sustainable cold-chain operation depends on timely monitoring at distributed nodes. Multi-sensor systems record temperature, relative humidity and gases to characterize handling conditions [1], and recent digital cold-chain frameworks combine these records with artificial intelligence for quality estimation and traceability [2,3]. Portable sensing nodes must nevertheless operate with restricted power and intermittent connectivity [4]. Environmental variables describe the conditions experienced by a batch but do not directly identify the ripeness or visible defects of individual products. This distinction is important in coffee supply chains, where harvesting and early grading influence subsequent processing, resource use and commercial value [5], while climate and disease pressures increase the need for timely product-state observations [6]. Camera-based inference at the cold-chain front end can therefore complement environmental sensing with localized information about coffee fruit maturity and visible quality.

Agricultural vision research has established crop-specific detection, sizing and maturity assessment across field and postharvest settings [7,8,9,10]. YOLO-based detectors, model pruning and integrated field prototypes have extended this progress toward real-time and on-device operation [11,12,13,14,15,16,17]. Efficient convolutional architectures provide compact feature extraction and cross-stage reuse [18,19,20,21,22], while attention-based designs offer a different balance between local computation and global interaction [23]. Hardware studies further show that operation counts alone do not determine energy use or accelerator efficiency [24,25,26]. These advances provide the technical basis for edge agricultural vision, but detector accuracy, robustness and hardware execution are commonly evaluated separately. Fine-grained coffee fruit assessment therefore lacks an evaluation that connects detector design, controlled visual degradation, NPU conversion and measured board-level energy efficiency.

This study addresses that gap through a detector architecture and evaluation protocol designed for low-power NPU execution. Distribution-based box regression can improve localization but adds output channels and integral operations [27]. Augmentation and corruption testing address different aspects of appearance variation [28,29,30]. Integer inference reduces arithmetic and memory costs only when the converted graph maps effectively to the target device [31,32]. The objective was to develop and evaluate an edge vision system that jointly considers fine-grained detection, controlled visual degradation, post-training quantization and measured board-level efficiency under the COCO evaluation convention [33]. The study provides four specific contributions:

1. A Branch-Integrated Feature Aggregation (BIFA) module combines a retained feature path with parallel convolutional paths to support discrimination among visually similar ripeness stages and complex backgrounds.
2. The YOLOv10-derived C2fCIB module is integrated into the YOLO11 feature-fusion neck to reduce redundant dense computation while preserving cross-stage feature reuse. Its contribution lies in its placement and coordinated use with BIFA and SRH within the proposed detector.
3. A Simplified Regression Head (SRH) replaces distribution-based regression with a compact convolutional output to reduce the deployment burden of NPU-supported 8-bit integer (INT8) inference.
4. The resulting system is evaluated through cross-architecture screening, progressive component ablation, four controlled visual-degradation tests, INT8 conversion and RK3576 measurements of throughput and board-input energy efficiency.

## 2. Related Work

### 2.1. Visual Sensing for Fruit Quality Assessment

Visual fruit assessment has progressed from general deep detectors to crop-specific sensing systems. DeepFruits established non-contact fruit detection for automated agricultural platforms [7]. Subsequent studies combined detection with branch segmentation for apple harvesting [8], RGB-D sensing for on-tree mango sizing [9], and augmented reality for strawberry-ripeness recognition [10]. YOLO-based detectors further enabled real-time fruit localization and load estimation [11,12], while pruning and task-specific adaptation reduced computational cost in agricultural scenes [13,16]. Coffee-specific studies have addressed ripeness detection under dense fruit, occlusion and variable backgrounds through lightweight YOLO variants, attention, adaptive sampling and hybrid convolution–transformer designs [34,35,36]. ASD-YOLO used a four-class Roboflow collection, OGS-YOLOv8 evaluated a separate five-class dataset, and SSViT-YOLOv11 combined public coffee-maturity images with independently collected data. Their dataset compositions and evaluation protocols differ from the six-class benchmark used here, so their results provide task context rather than controlled numerical baselines. Reviews and integrated field prototypes indicate growing interest in on-device agricultural detection, but also reveal heterogeneous evaluation and deployment practices [14,15]. These studies demonstrate the value of visual sensing in agricultural workflows, although evidence remains fragmented across datasets, class definitions and deployment settings.

### 2.2. Efficient Detector Architectures

Efficient detector design combines compact feature extraction with selective feature reuse. Among recent one-stage detectors, the YOLO11 family includes the lightweight YOLO11n variant [17]. MobileNetV2, MobileNetV3 and GhostNet reduce dense computation through inverted residual, lightweight search and inexpensive feature-generation mechanisms [18,19,20]. CSPNet improves cross-stage feature reuse [21], while YOLOv10 introduced the Compact Inverted Block (CIB) and C2fCIB configuration for efficient spatial and channel mixing [22]. Attention-based detectors provide strong global interaction but rely on matrix operations whose compilation and scheduling may be less predictable on resource-constrained NPUs [23]. Taken together, these studies show that an embedded detector must balance representational capacity, computational economy and compatibility with the target accelerator. However, the reviewed architectures do not by themselves resolve how fine-grained ripeness representation, multi-scale feature fusion and NPU-compatible regression should be coordinated within an embedded coffee-fruit detector.

### 2.3. Edge Deployment and Robustness Evaluation

Deployment efficiency depends on the interaction between model structure, numerical precision and hardware execution. Energy use is influenced by memory movement, processor organization and operator mapping, so theoretical operation counts cannot replace device-level measurements [24,25,26]. Distribution-based box regression can improve localization but adds output channels and integral operations [27]. Data augmentation and corruption testing address different aspects of appearance variation [28,29,30], while integer inference reduces arithmetic and memory costs only when the converted graph maps effectively to the target device [31,32]. Evaluation under the COCO convention provides standardized detection metrics [33], but embedded suitability additionally requires conversion fidelity, throughput and measured energy efficiency. This combination of accuracy, robustness and physical deployment evidence defines the evaluation scope adopted in this study.

## 3. Methodology

**Figure 1:**
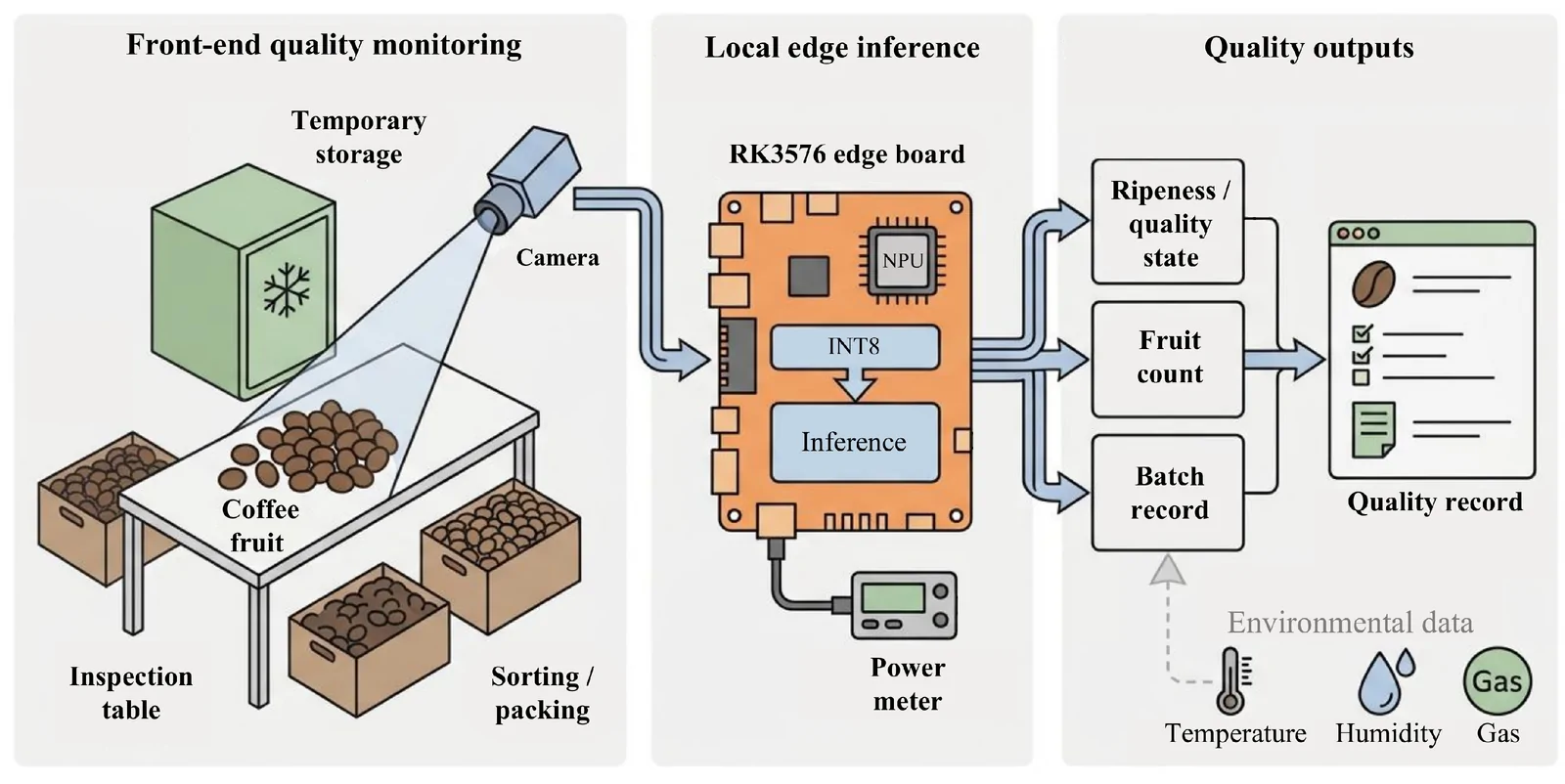
Conceptual deployment context for camera-based front-end cold-chain quality monitoring. The inspection table illustrates a downstream grading and packing use case, whereas the benchmark images are acquired directly from coffee fruits on the tree, as represented in Figure 2.

The proposed vision node links tree-side quality observation with downstream postharvest handling. A camera acquires coffee-fruit images directly from the tree, and the embedded device identifies ripeness and visible-quality classes locally. The resulting class labels, confidence scores, object counts and timestamps can accompany later grading, temporary storage and sorting records. Figure 1 defines this operational boundary: the inspection-table and packing elements represent a downstream grading and packing use case, whereas the benchmark images depict coffee fruit on the tree. Environmental variables are treated as contextual records rather than model inputs. The evaluation focuses on tree-side visual detection and embedded execution, whereas multimodal sensor fusion and physical transport operations are outside its scope.

**Figure 2:**
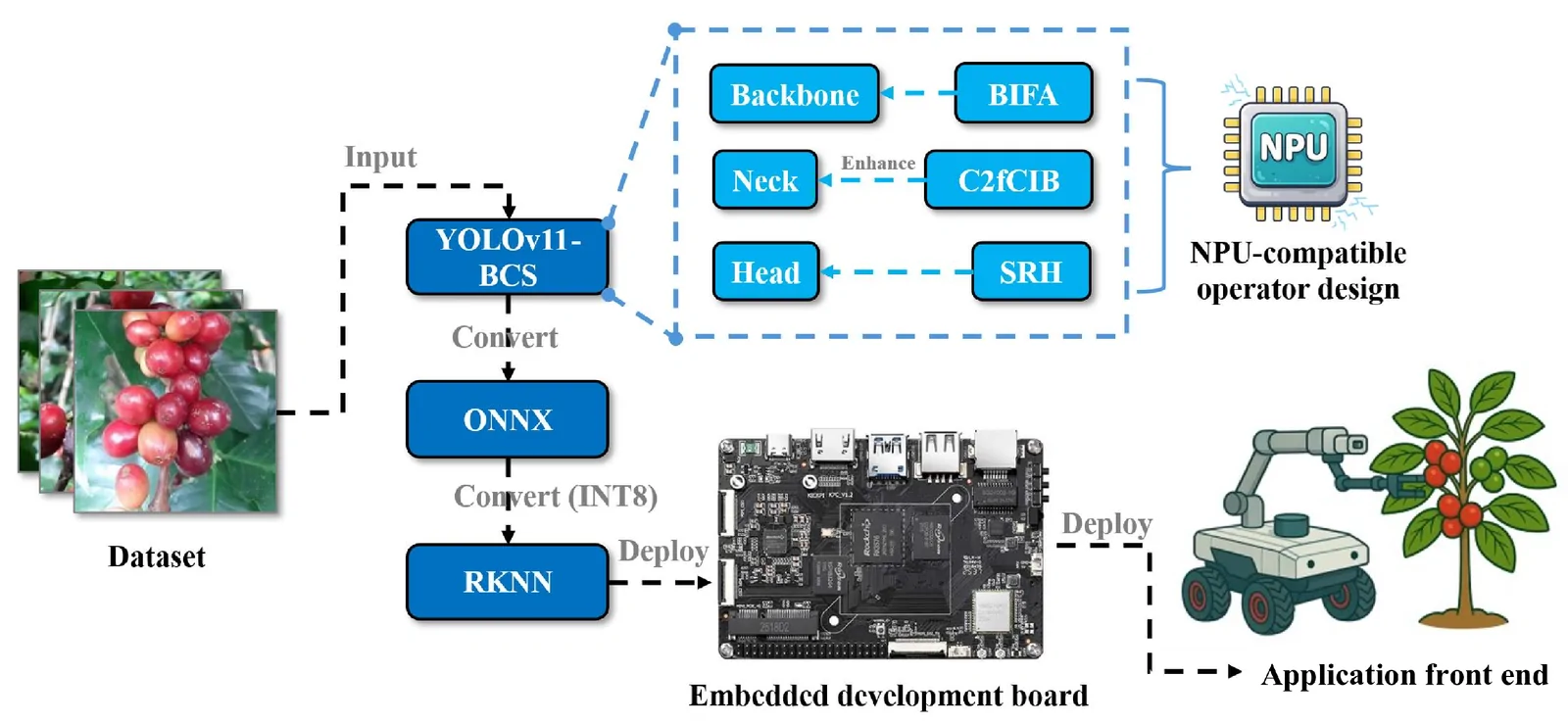
Tree-side image input, YOLO11BCS conversion and edge-deployment workflow for front-end cold-chain quality monitoring.

Within this system boundary, a tree-side camera frame is converted into a structured quality record under the computational constraints of the edge platform. Figure 2 summarizes the corresponding image-to-device path and shows the tree-side coffee-fruit imagery used as the visual input context. Model training and structural optimization produce YOLO11BCS, which is exported to Open Neural Network Exchange (ONNX), converted by the Rockchip Neural Network (RKNN) toolkit and executed as an 8-bit integer (INT8) model on the RK3576 neural processing unit (NPU). Image preprocessing and detection postprocessing run on the central processing unit (CPU), whereas the network forward pass runs on the NPU. Thus, Figure 1 describes the broader application context, while Figure 2 specifies the evaluated tree-side image input and its computational implementation.

The methodology follows the information path from image acquisition to embedded quality output. Dataset preparation and preprocessing define the basis for model comparison. The detector architecture then combines fine-grained feature representation with operators compatible with the target NPU. Finally, quantization, controlled degradation tests and board-input power measurements evaluate accuracy and execution behavior at the device level.

### 3.1. Dataset, Partitioning and Preprocessing

The dataset defines the visual variability represented in model development and evaluation. The evaluated files were obtained from the public “coffee cherry” object-detection project on Roboflow Universe under a Creative Commons Attribution (CC BY) 4.0 license [37]. Its 1639 exported images comprise 1339 training, 200 validation and 100 test files derived from 996 source images. The associated Coffee Fruit Maturity resource describes the fruit as *Coffea arabica* and provides five maturity-stage labels [38]. The image set contains natural scenes with changing illumination, foliage and branch occlusion, isolated and densely clustered fruit, varied camera orientations and both close and wider orchard backgrounds. Image-level metadata do not document cultivar, acquisition site, season, camera model or environmental measurements, so the data do not support stratified claims across those factors.

The export contains five maturity-stage labels—unripe, semi-ripe, ripe, overripe and dry—and a separate diseased class for visible fruit condition. Coffee quality guidance describes unripe cherries as green, semi-ripe cherries as mixtures of green and red or yellow, ripe cherries as fully red or yellow, depending on cultivar, and overripe cherries as darkening and beginning to wrinkle [39]. These descriptors aid interpretation but were not the dataset annotation protocol. All six classes occur in each partition, although unripe fruit constitutes 73.9% of the 24,005 bounding boxes. The class names and bounding-box annotations are those distributed with the public dataset and were not redefined for this study. The public records do not identify the annotators, report agreement or formal thresholds for dry and diseased fruit, or document expert validation. The labels are therefore treated as dataset-provided operational categories rather than as a newly validated physiological or pathological grading standard.

**Figure 3:**
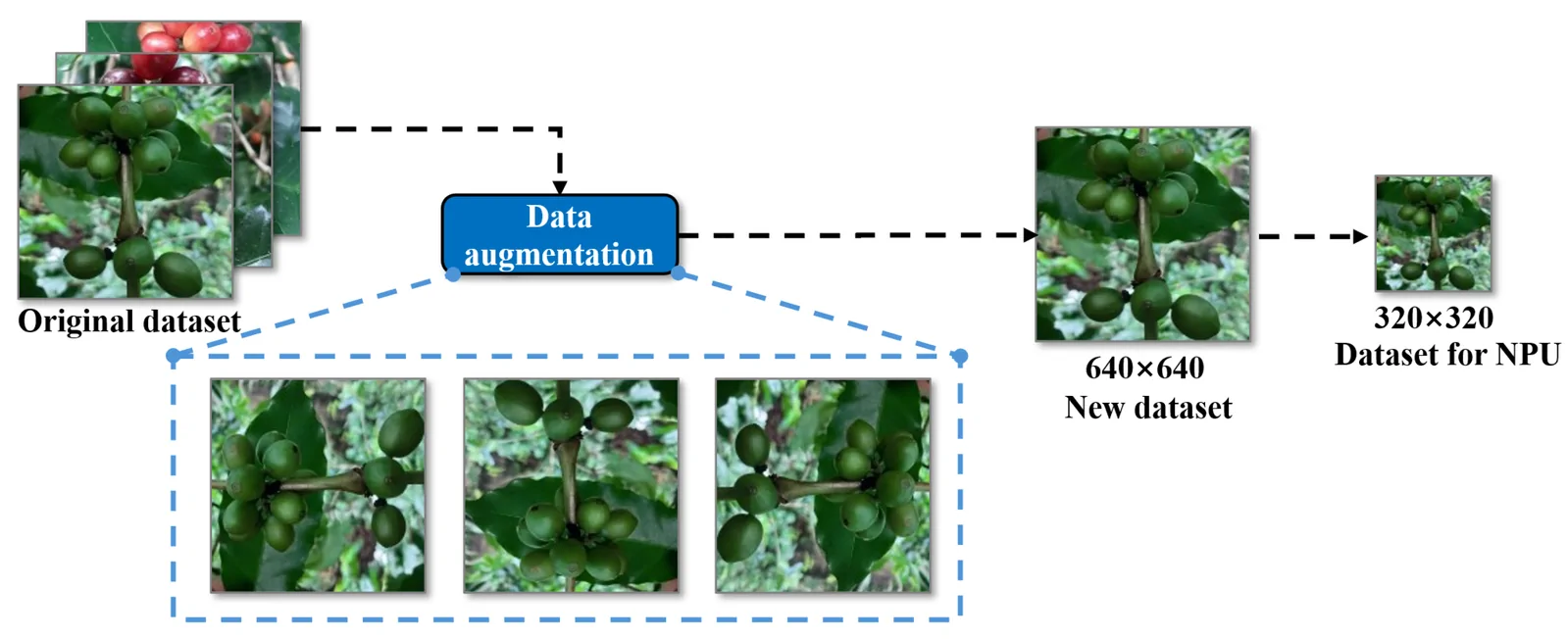
Source-image partitioning, training augmentation and input-size adjustment for the coffee-fruit dataset.

The dataset documentation describes an approximately 7:2:1 source-image split. The experiments followed the supplied training, validation and test partitions, which contained 1339 training images, 200 validation images and 100 test images derived from 996 source images. Transformed versions of some source images occur in more than one partition, involving 130 training–validation, 63 training–test and 12 validation–test source-image correspondences. The supplied split therefore does not establish source-image or plant-level independence, and the reported detection metrics characterize performance within this public benchmark rather than independent field generalization. During model fitting, the training pipeline applied rotation, spatial transformations, and brightness, contrast and saturation adjustments, whereas evaluation used the fixed validation and test partitions without online augmentation. All images were resized to 320×320 pixels for the main experiments because the lower spatial workload is relevant to embedded throughput and memory use, while the 640×640 setting was retained to quantify the accuracy–efficiency trade-off. Photometric augmentation was used as a training regularizer [28,29,30]. Because color is also an informative maturity cue [39], photometric transformations can improve tolerance to illumination variation while altering information relevant to the class labels. No separate expert relabeling or physiological validation of the transformed colors was performed. The aggregate effect of the complete augmentation policy is reported in Section 4.1. Individual transformation effects and the physiological validity of transformed color cues were not evaluated separately. Figure 3 shows the resulting workflow.

### 3.2. YOLO11BCS Detector

#### 3.2.1. Architecture and Hardware-Oriented Design

**Figure 4:**
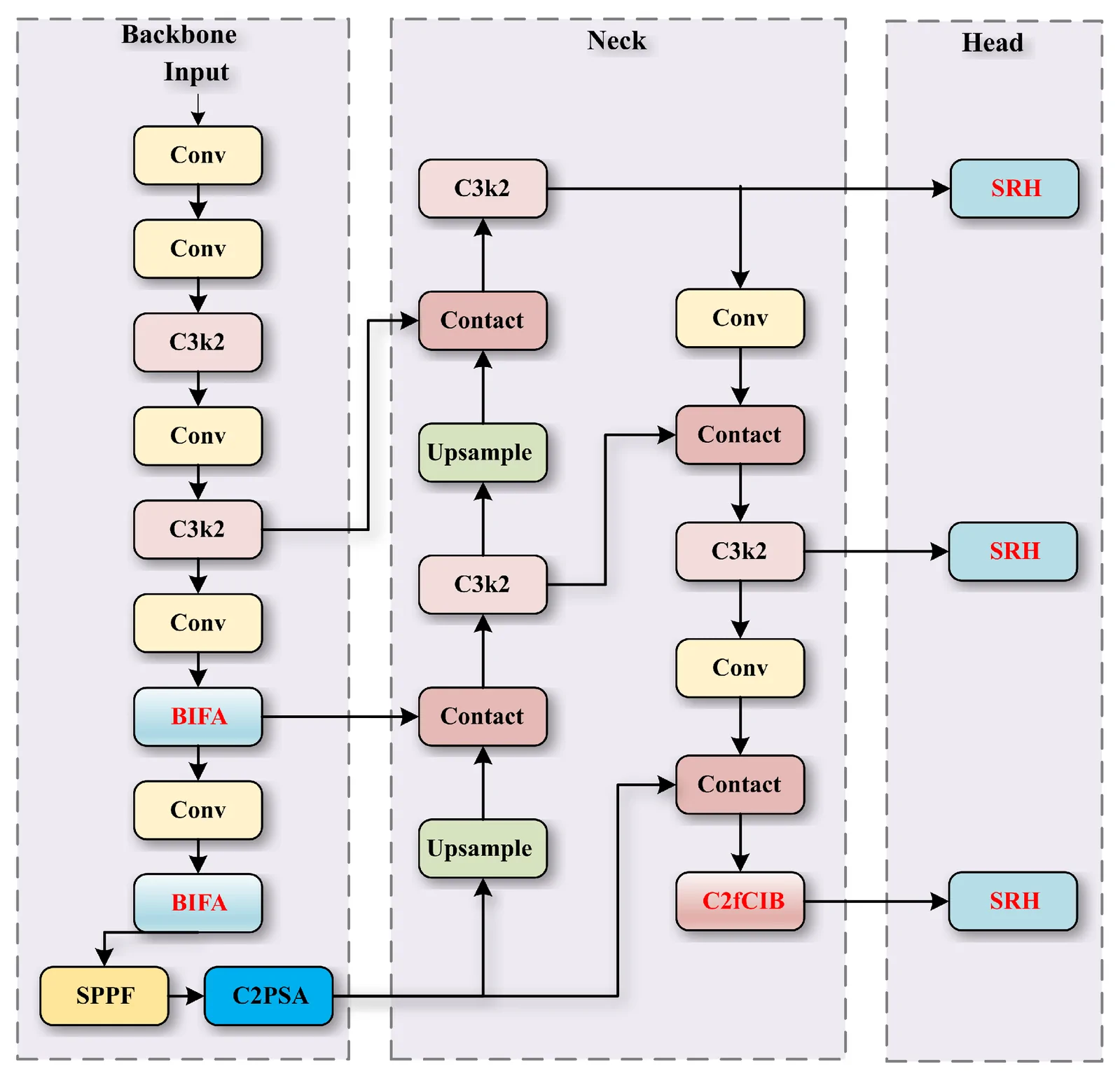
YOLO11BCS architecture, including the BIFA backbone module, C2fCIB feature-fusion module and SRH regression head.

The deployment architecture must combine task-specific localization with a computation path suitable for low-power inference. The proposed detector, YOLO11BCS, therefore uses the lightweight YOLO11n model as its starting point. The YOLO paradigm performs full-image detection in a single-stage network and was originally developed to support end-to-end, real-time inference [11]. Its practical suitability for agricultural vision is supported by real-time fruit-detection studies and broader reviews of YOLO-based monitoring, sensing and automation [12,13,14,16]. More recent efficiency-oriented YOLO research also shows that reducing computational redundancy within the backbone, neck and detection head can improve the performance–latency balance [22]. These findings provide the family-level rationale, whereas the choice of YOLO11n was determined by the task-specific screening in Section 4.1. At 640×640 without augmentation, YOLO11n achieved 75.7% mAP50 and the highest mAP50:95 among the evaluated YOLO candidates, reaching 70.0%. It also exceeded the evaluated RT-DETR and STN-YOLO configurations by 4.0 and 6.2 percentage points in mAP50:95, respectively. Structurally, its convolutional multi-scale hierarchy supports fruits with different apparent sizes, and its modular backbone, neck and head allow targeted changes without replacing the complete execution graph. This modularity is important because visually similar ripeness classes require stronger local representation, while the RK3576 path favors convolution, concatenation and element-wise operations supported by the RKNN toolchain. Device efficiency cannot be inferred from the operation count alone because memory movement, processor organization and operator mapping also affect execution [24,25,26]. RKNN-Toolkit2 provides conversion, quantization and target-platform evaluation within its supported operator set [40]. As shown in Figure 4, BIFA replaces the two C3k2 blocks at the P4/16 and P5/32 backbone stages, and C2fCIB replaces the final C3k2 fusion block on the P5/32 neck path. SRH is applied to all three P3/8, P4/16 and P5/32 detection scales. These placements assign BIFA, C2fCIB and SRH to local representation, multi-scale fusion and regression execution, respectively, while retaining the selected single-stage detection framework.

#### 3.2.2. Branch-Integrated Feature Aggregation

Fine-grained fruit discrimination depends on retaining both low-level appearance cues and locally transformed responses. BIFA addresses this requirement with a branch–transform–merge structure that preserves a direct feature path while applying local filtering to two complementary paths. For an input feature map, $X_{\mathrm{in}}\in R^{C\times H\times W}$, where *C*, *H* and *W* denote the channel number, height and width, respectively, a 1×1 convolution first performs channel compression and cross-channel interaction:

(1) $$X_{\mathrm{mid}}=\mathrm{SiLU}\;\left(\mathrm{BN}\;\left({\mathrm{Conv}}_{1\times 1}\left(X_{\mathrm{in}}\right)\right)\right).$$

**Figure 5:**
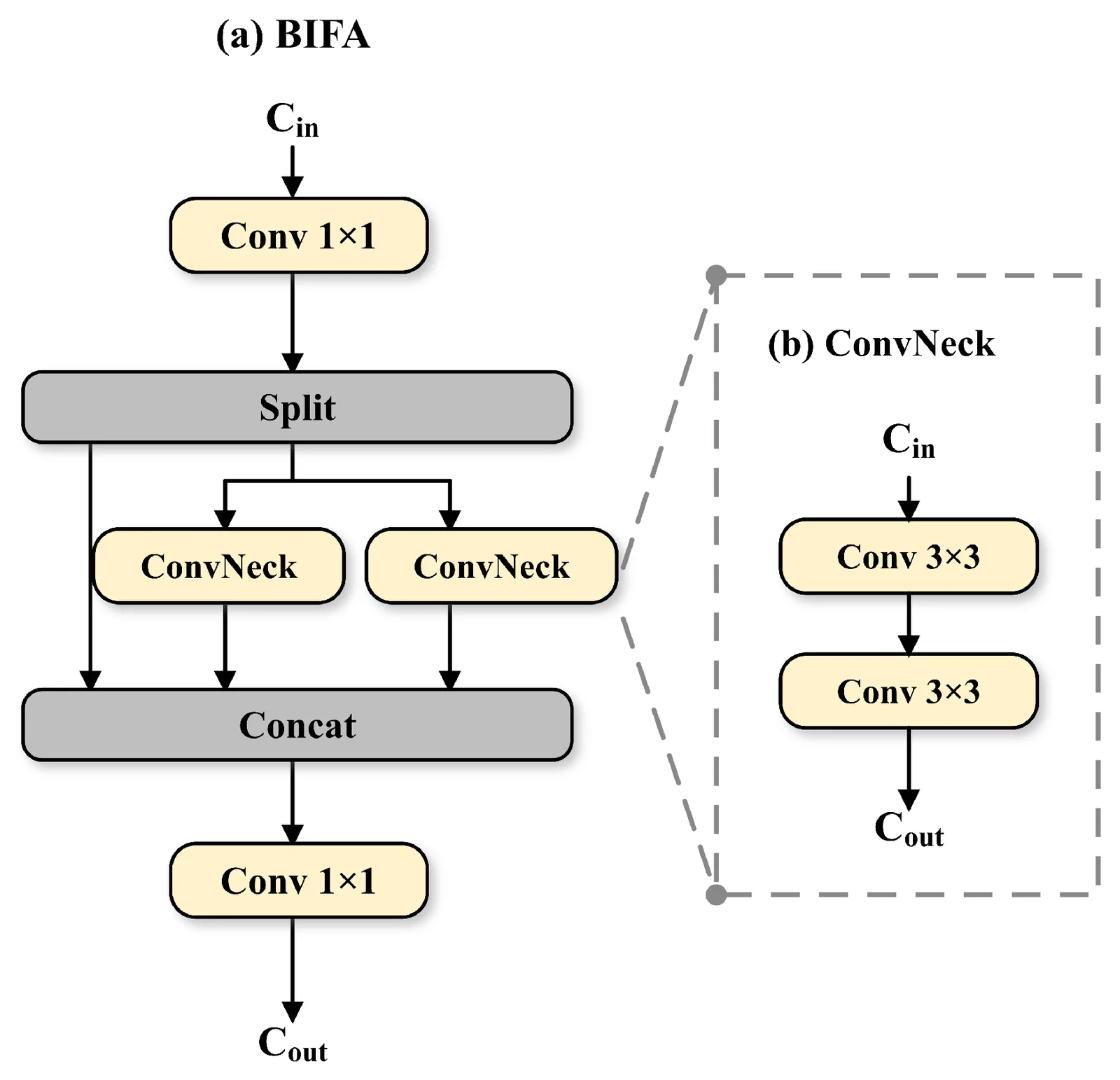
BIFA module and ConvNeck submodule.

Here, ${\mathrm{Conv}}_{1\times 1}$ is a pointwise convolution, BN is batch normalization, SiLU is the sigmoid linear unit, and $X_{\mathrm{mid}}$ is the compressed intermediate feature. The pointwise projection limits the channel cost before branching, while the nonlinear activation preserves feature selectivity after normalization. The intermediate representation is then routed through the three functional paths denoted by the Split block in Figure 5:

(2) $$\left[X_{0},X_{1},X_{2}\right]={\mathrm{Branch}}_{c}\left(X_{\mathrm{mid}}\right),\;C_{0}=C_{1}=C_{2}=\frac{C_{\mathrm{mid}}}{2},$$

where ${\mathrm{Branch}}_{c}$ denotes the functional channel allocation shown in Figure 5, $C_{0}$, $C_{1}$ and $C_{2}$ are the effective output widths of the retained and two transformed paths, and $C_{\mathrm{mid}}$ is the channel number of $X_{\mathrm{mid}}$. In the evaluated nano-scale configuration, the P4/16 BIFA block uses $C_{\mathrm{in}}=C_{\mathrm{out}}=C_{\mathrm{mid}}=128$ and $\left(C_{0},C_{1},C_{2}\right)=\left(64,64,64\right)$, whereas the P5/32 block uses $C_{\mathrm{in}}=C_{\mathrm{out}}=C_{\mathrm{mid}}=256$ and $\left(C_{0},C_{1},C_{2}\right)=\left(128,128,128\right)$. These values describe the three principal paths shown in Figure 5; residual feature propagation within the underlying cross-stage block remains unchanged. The retained path preserves the compressed input response, while the other two paths are processed independently by ConvNeck to provide complementary local transformations:

(3) $$Y_{1}=\mathrm{ConvNeck}\left(X_{1}\right),\;Y_{2}=\mathrm{ConvNeck}\left(X_{2}\right),$$

(4) $$\mathrm{ConvNeck}\left(x\right)={\mathrm{Conv}}_{3\times 3}\;\left({\mathrm{Conv}}_{3\times 3}\left(x\right)\right),$$

where *x* is a branch input, $Y_{1}$ and $Y_{2}$ are the corresponding branch outputs, and ${\mathrm{Conv}}_{3\times 3}$ denotes a 3×3 convolution. The retained path bypasses the ConvNeck operations, whereas the two transformed paths provide additional local filtering. All three paths are then concatenated and projected:

(5) $$X_{\mathrm{out}}={\mathrm{Conv}}_{1\times 1}\;\left(\mathrm{Concat}(X_{0},Y_{1},Y_{2})\right),$$

where Concat concatenates features along the channel dimension, and $X_{\mathrm{out}}$ is the output feature. The final pointwise projection restores the required output-channel interface after concatenation. Thus, the retained path preserves the compressed input response, while the two parallel convolutional paths enlarge local receptive-field coverage without introducing attention or dynamic routing. The module is illustrated in Figure 5.

#### 3.2.3. Cross-Stage Partial Network with Compact Inverted Block

**Figure 6:**
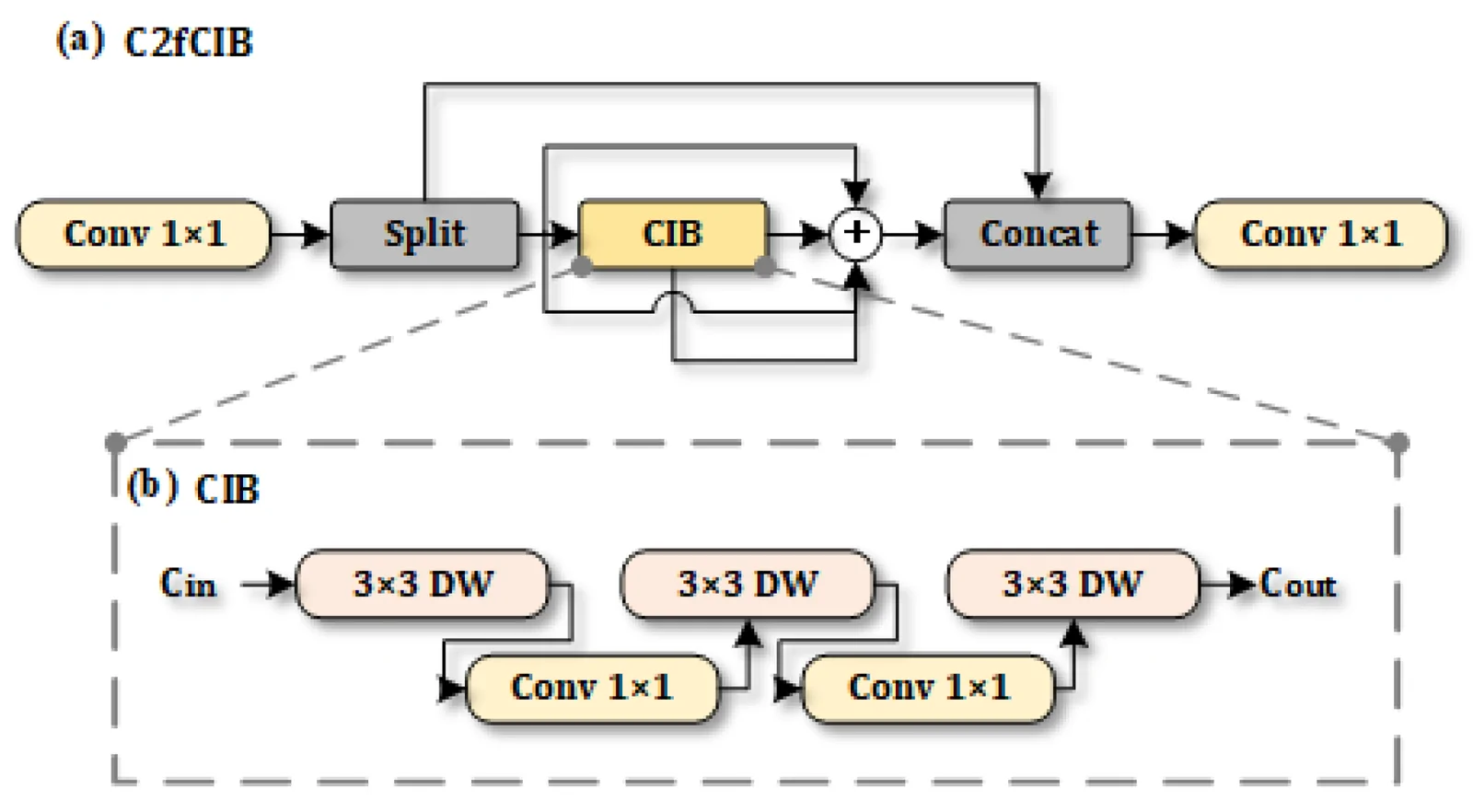
C2fCIB module and compact inverted block.

Multi-scale fusion must exchange useful features without imposing the cost of repeated dense convolutions. The C2fCIB used in this study is derived from YOLOv10, which introduced CIB within a cross-stage partial structure for efficient spatial and channel mixing [22]. Its inner sequence of three depthwise and two pointwise convolutions is inherited from that architecture. The present configuration places C2fCIB at the selected YOLO11 neck stage shown in Figure 4 and coordinates it with BIFA in the backbone and SRH in the detection head. This placement preserves a direct cross-stage feature route while reducing dense-convolution redundancy during feature fusion. The macroblock combines split paths, residual addition and concatenation, as shown in Figure 6. Within the CIB path, depthwise convolutions perform spatial filtering and pointwise convolutions mix channels. Its operator-level description is:

(6) $$F_{1}={\mathrm{DWConv}}_{3\times 3,1}\left(F_{\mathrm{in}}\right),\;F_{2}={\mathrm{PWConv}}_{1}\left(F_{1}\right),$$

followed by

(7) $$F_{3}={\mathrm{DWConv}}_{3\times 3,2}\left(F_{2}\right),\;F_{4}={\mathrm{PWConv}}_{2}\left(F_{3}\right),\;F_{\mathrm{out}}={\mathrm{DWConv}}_{3\times 3,3}\left(F_{4}\right),$$

where $F_{\mathrm{in}}$ is the input feature to the CIB path, ${\mathrm{DWConv}}_{3\times 3,i}$ denotes the *i*th 3×3 depthwise convolution, ${\mathrm{PWConv}}_{i}$ denotes the *i*th 1×1 pointwise convolution, $F_{1}$–$F_{4}$ are intermediate features, and $F_{\mathrm{out}}$ is the CIB output. These equations describe the inherited inner CIB sequence. The surrounding split, residual and concatenation operations form the C2fCIB macroblock shown in Figure 6. The evaluated contribution is the placement of this block within the proposed YOLO11-based configuration and its coordinated use with BIFA and SRH.

#### 3.2.4. Simplified Regression Head

**Figure 7:**
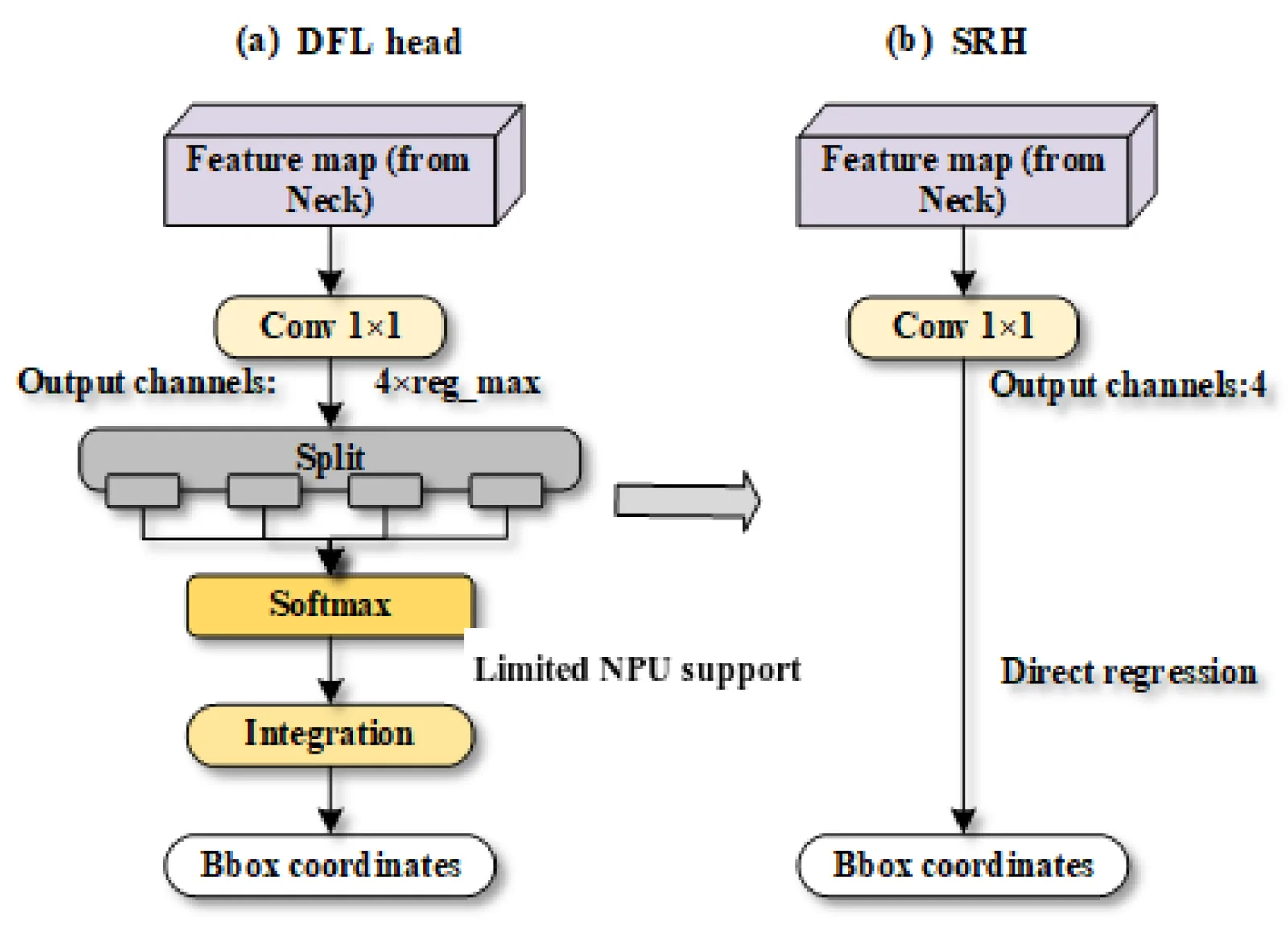
Comparison of the standard DFL regression head and SRH.

The regression head determines how feature-map responses are converted into bounding-box coordinates, so its output representation directly affects both localization and deployment cost. The standard YOLO11 head uses Distribution Focal Loss (DFL) to represent a distribution over box distances [27]. This representation requires a wider output tensor and distribution decoding. SRH is a reduced regression-channel configuration that sets the regression-channel parameter to one and directly outputs four box offsets:

(8) $$B={\mathrm{Conv}}_{1\times 1}\left(X_{\mathrm{feat}}\right)\in R^{4\times H\times W},$$

where $X_{\mathrm{feat}}$ is the input feature to the head, $B=(b_{l},b_{t},b_{r},b_{b})$ is the four-channel map of left, top, right and bottom offsets, and *H* and *W* are its spatial dimensions. Training uses Complete Intersection over Union (CIoU) loss for box regression and binary cross-entropy loss for classification; the DFL term is disabled when the regression-channel parameter is one. CIoU is based on the intersection over union (IoU), the squared distance between predicted and ground-truth box centers, the diagonal length of the smallest enclosing box, and an aspect-ratio term [41]. The direct four-offset representation reduces the distribution-output burden and reliance on operations with weaker NPU support, while CIoU retains localization supervision during training. Figure 7 compares SRH with the standard DFL head.

### 3.3. Training and Evaluation Protocol

The comparative protocol fixes the nominal training budget, evaluation thresholds and hardware before model evaluation. All models use Ultralytics YOLO version 8.3.63 with the PyTorch deep-learning framework on an NVIDIA GeForce RTX 5060 Ti GPU with 16 GB of memory (NVIDIA Corporation, Santa Clara, CA, USA), 300 training epochs, a batch size of 64 and a random seed of 0 with deterministic execution enabled. The networks are initialized from their model definitions without pretrained weights. Optimization uses AdamW with an initial learning rate of 0.001, a first-moment coefficient of 0.9 and a weight decay of 0.0005. After three warm-up epochs, a linear schedule reduces the learning rate to 1% of its initial value by the final epoch. The evaluator uses a confidence threshold of 0.001 and a non-maximum suppression (NMS) IoU threshold of 0.6. The input size is set to 320×320 or 640×640, according to the comparison. Server-side throughput is measured on an NVIDIA Tesla T4 GPU (NVIDIA Corporation, Santa Clara, CA, USA), and embedded execution is evaluated on the RK3576 system-on-chip (Rockchip Electronics Co., Ltd., Fuzhou, China). Each reported configuration is represented by one completed training run under fixed data partitions, software environment, hardware, nominal schedule and evaluation thresholds. These controls support matched comparisons across configurations. The accuracy values remain descriptive point estimates because the protocol does not quantify run-to-run variability or inferential significance. In the ablation and deployment analyses, YOLO11 refers to the lightweight YOLO11n baseline evaluated in Section 4.1.

The detection metrics are defined to separate overlap quality, the retrieval of labeled objects and localization across operating thresholds. For a predicted box, $B_{p}$, and a ground-truth box, $B_{g}$, their intersection over union (IoU) is

(9) $$\mathrm{IoU}\left(B_{p},B_{g}\right)=\frac{|B_{p}\cap B_{g}|}{|B_{p}\cup B_{g}|}.$$

At an IoU threshold, τ, a matched prediction is counted as a true positive (TP), an unmatched prediction as a false positive (FP), and an unmatched ground-truth object as a false negative (FN). Precision and recall are then calculated as

(10) $$P\left(\tau \right)=\frac{\mathrm{TP}(\tau )}{\mathrm{TP}(\tau )+\mathrm{FP}(\tau )},\;R\left(\tau \right)=\frac{\mathrm{TP}(\tau )}{\mathrm{TP}(\tau )+\mathrm{FN}(\tau )}.$$

For class *c*, ${\mathrm{AP}}_{c}\left(\tau \right)$ is the area under the precision–recall curve at threshold τ, and the mean average precision over the C=6 classes is

(11) $${\mathrm{mAP}}_{\tau }=\frac{1}{C}\sum_{c=1}^{C}{\mathrm{AP}}_{c}\left(\tau \right).$$

Accordingly, ${\mathrm{mAP}}_{50}={\mathrm{mAP}}_{0.50}$, whereas the stricter ${\mathrm{mAP}}_{50:95}$ is the mean over ten IoU thresholds:

(12) $${\mathrm{mAP}}_{50:95}=\frac{1}{10}\sum_{k=0}^{9}{\mathrm{mAP}}_{0.50+0.05k}.$$

Here, *k* indexes the ten IoU thresholds from 0.50 to 0.95. These definitions follow the COCO evaluation convention [33]. The reported table columns P and R are obtained from the same evaluation pipeline for all compared models, while mAP50 and mAP50:95 summarize the corresponding precision–recall areas across the stated IoU thresholds.

The system metrics connect model complexity with measured execution and energy use. Computational complexity is reported in giga floating-point operations (GFLOPs) at 640×640 unless a different resolution is stated explicitly. Speed is reported as frames per second (FPS), and board-level energy efficiency is calculated as

(13) $$\eta_{E}=\frac{\mathrm{FPS}}{P_{\mathrm{board}}},$$

where $\eta_{E}$ is FPS/W, and $P_{\mathrm{board}}$ is the mean RK3576 board-input power in watts. The external meter captures the board during steady-state execution, including host-side preprocessing and postprocessing, data transfer and NPU inference, but not the power of an externally supplied camera, display, communication link or upstream power-supply losses. Tesla T4 power is obtained from the NVIDIA Management Library (NVML), whereas RK3576 power is measured at the board input. The two measurements therefore have different system boundaries and are not compared directly.

### 3.4. Quantization, Embedded Deployment and Power Measurement

The deployment configuration is chosen to preserve the trained detector while making its execution compatible with the target NPU. After training, the model is converted from PyTorch to ONNX and then to RKNN. RKNN-Toolkit2 is used for conversion, quantization and performance evaluation, whereas RKNN-Toolkit-Lite2 invokes the deployed model on the target board. The deployment uses INT8-oriented hybrid post-training quantization, which reduces arithmetic and memory-transfer cost without retraining the detector. The six output tensors associated with the three detection scales are assigned to independently configurable hybrid subgraphs because their activation distributions can differ across scales. Separate settings avoid imposing one calibration range on all detection outputs. The calibration set consists of the first 100 images from the training partition, so calibration samples are drawn from the training distribution without using validation or test images. Quantization thresholds are selected using the Kullback–Leibler (KL) divergence criterion to limit the distributional discrepancy between pre- and post-quantization activations, and per-channel quantization is applied to retain channel-specific scales [31,32]. The main settings are summarized in Table 1.

The RK3576 system-on-chip combines an eight-core CPU, a Mali-G52 MC3 GPU and a 6-TOPS NPU, with a 32-bit LPDDR4/LPDDR4X/LPDDR5 memory interface [42]. In the evaluated execution path, host-side preprocessing and postprocessing run on the CPU, whereas INT8 detector inference runs on the NPU through RKNN-Toolkit-Lite2. For the YOLO11 baseline, the NPU returns quantized distribution logits from the three detection scales. The CPU then applies softmax normalization and expectation over the DFL bins before reconstructing box coordinates, filtering candidates and applying NMS. SRH instead returns four direct offsets per spatial location, so its CPU postprocessing omits the DFL-specific softmax and expectation while retaining the same coordinate mapping, confidence threshold and NMS threshold. Both models use the same INT8 calibration data, preprocessing procedure and end-to-end timing boundary.

Throughput was measured from image input to detection output during continuous inference and therefore includes host preprocessing, data transfer, NPU inference and host postprocessing. Throughput alone does not determine the energy cost of embedded inference, so power was measured at the board input rather than inferred from a nominal device specification. A ZLG PA310 direct-current power meter (Guangzhou Zhiyuan Electronics Co., Ltd., Guangzhou, China) was connected between the external supply and the RK3576 board and synchronously sampled voltage and current at 500 kS/s. After a 20 min warm-up to reduce thermal and frequency-transient effects, mean active power was recorded over 60 s of steady-state inference. Each configuration was measured in three independent 60 s runs, and power is reported as the mean ± sample standard deviation across the three run-level means. FPS/W was calculated from mean board-input power and image-to-detection-output throughput. The measurement boundary did not include the power of an externally supplied camera, display or communication equipment, nor did it characterize standby operation. The reported energy metrics therefore quantify the RK3576 execution subsystem under continuous inference rather than the total energy demand of a deployed cold-chain installation.

### 3.5. Robustness Test Design

Robustness evaluation must perturb image quality without changing object identity or label geometry. To represent image-quality changes that may occur with compact cameras, platform motion and outdoor handling, four degradations are applied to the clean test images: Gaussian noise, illumination variation, motion blur and rain. The original labels are retained, and no model is retrained on the degraded images, so differences across severity levels reflect the response of the fixed detectors to the specified image transformations. The transformations are defined as

(14) $$\begin{array}{cccc} I_{\mathrm{gauss}}\left(x,y\right) & =I\left(x,y\right)+N\left(0,\sigma^{2}\right), & \sigma & \in \{10,20,30\}, \end{array}$$

(15) $$\begin{array}{cccc} I_{\mathrm{illum}}\left(x,y\right) & =\beta I(x,y), & \beta & \in \{0.7,0.5,0.3\}, \end{array}$$

(16) $$\begin{array}{cccc} I_{\mathrm{blur}}\left(x,y\right) & =K_{l,\theta }*I\left(x,y\right), & l & \in \{7,13,21\}, \end{array}$$

(17) $$\begin{array}{cccc} I_{\mathrm{rain}}\left(x,y\right) & =(1-\alpha )I(x,y)+\alpha R(x,y), & \alpha & \in \{0.15,0.22,0.30\}. \end{array}$$

**Figure 8:**
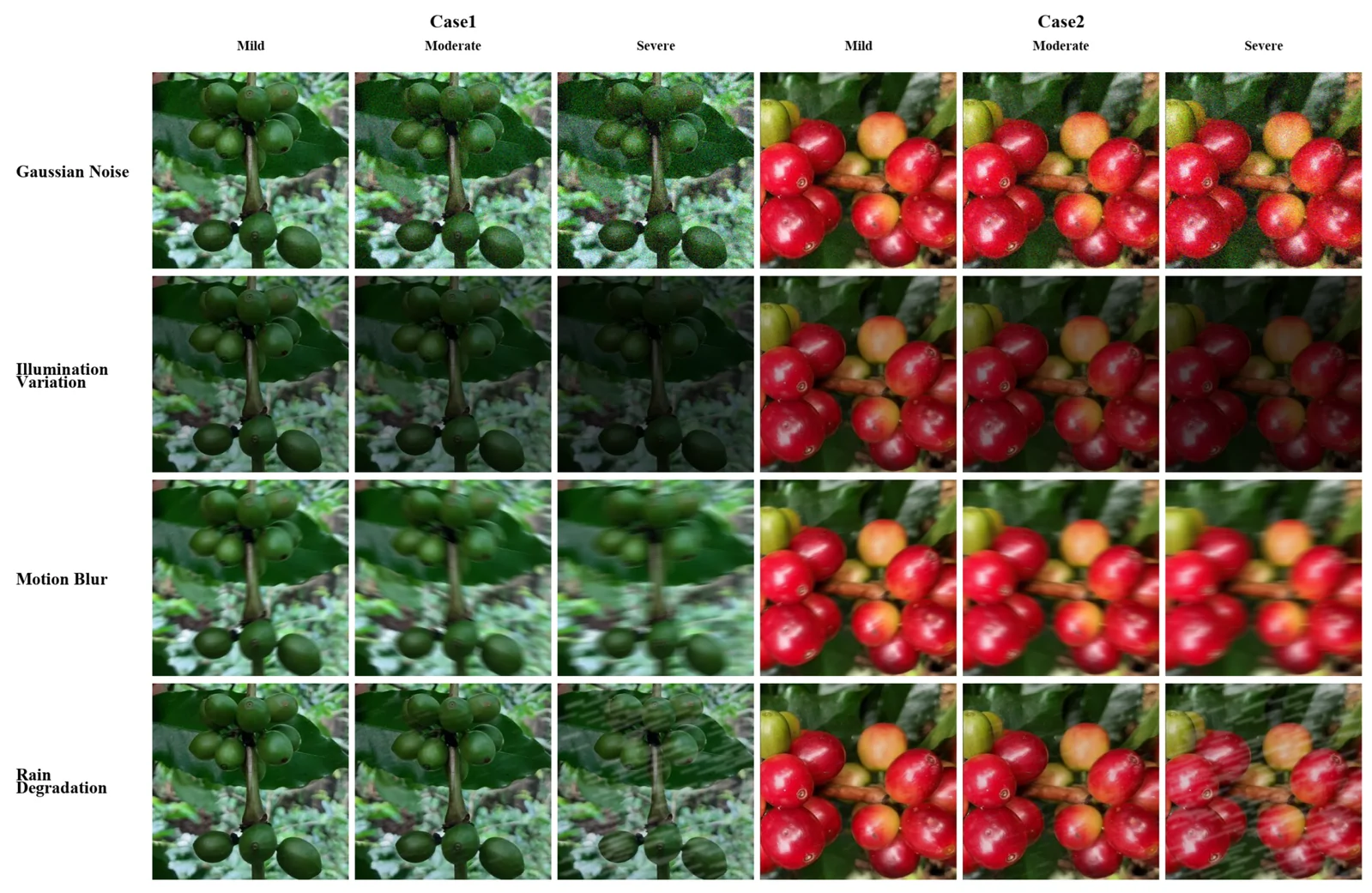
Representative samples of four synthetic visual degradations at mild, moderate and severe levels.

Here, I(x,y) is the original pixel value at coordinate (x,y), and $N(0,\sigma^{2})$ is zero-mean Gaussian noise with standard deviation σ. The brightness-scaling factor is denoted by β. The term $K_{l,\theta }$ represents a linear motion-blur kernel of length *l* and direction θ, while R(x,y) denotes the synthetic rain field. The rain-field mixing coefficient is α, and * denotes convolution. The blur direction is sampled from $\left[-25^{\circ },25^{\circ }\right]$ to represent variable relative motion. The rain field uses a fixed intensity value of 235 and a direction of $-18^{\circ }$. Its density, streak length, width, blur radius and brightness factor are varied jointly across the three levels to increase the strength of the overlay. The mild, moderate and severe densities are 0.0010, 0.0018 and 0.0030, respectively. The corresponding streak lengths are 14, 22 and 30 pixels, the widths are 1, 1 and 2 pixels, the blur radii are 1.1, 1.5 and 2.0 pixels, and the brightness factors are 0.95, 0.90 and 0.85. The three severity levels follow the graded-corruption principle used in common robustness benchmarks [43]. The complete parameter combinations are provided in Supplementary Table S1. Representative samples are shown in Figure 8.

## 4. Results and Discussion

The results follow four linked questions: baseline selection, component-level changes in accuracy and complexity, sensitivity to controlled visual degradation, and performance after conversion to the RK3576 deployment path. This sequence separates architecture screening from matched ablation and then connects model-level results to device-level measurements.

### 4.1. Baseline Selection and Cross-Architecture Comparison

Lightweight YOLO candidates are first screened under the 640×640 non-augmented setting using the same data partition and nominal training schedule. Table 2 presents this architecture-selection stage. YOLO11n and YOLOv13n have nearly identical mAP50 values, 75.7% and 75.6%, respectively, but YOLO11n obtains the highest mAP50:95 at 70.0%, compared with 69.6% for YOLOv13n. The 0.4-percentage-point difference is small, but it occurs in the stricter metric that averages localization quality over multiple IoU thresholds. Because different architectures may converge at different rates, equal epoch counts do not establish equivalent optimization exposure. Accordingly, the table provides descriptive evidence for selecting the starting architecture, while the final architectural effect is estimated from the matched YOLO11 lineage in Section 4.2.

The selected baseline is then screened against representative detector families beyond YOLO under the same 640×640 non-augmented setting. Table 3 includes the two-stage convolutional detector Faster R-CNN [44], the dense one-stage detector RetinaNet [45], SSD with a MobileNetV3 backbone [19], the transformer-based Real-Time DEtection TRansformer (RT-DETR) [46], and the agricultural STN-YOLO model [47]. RT-DETR obtains the highest precision at 73.9%, but its mAP50:95 is 66.0%, 4.0 percentage points below YOLO11n. STN-YOLO reaches a comparable mAP50 of 75.3% but a lower mAP50:95 of 63.8%. These screening values provide descriptive cross-family context under the 640×640 non-augmented setting. The matched YOLO11 lineage in Section 4.2 provides the controlled estimate of the final architectural effect under the 320×320 augmented protocol.

After the baseline architecture has been selected, Table 4 evaluates the effects of training augmentation and input size on YOLO11 performance and Tesla T4 throughput. This comparison selects the input configuration used for the subsequent structural ablation and embedded evaluation.

Training with the complete augmentation policy at 640×640 increases mAP50 from 75.7% to 84.2% and mAP50:95 from 70.0% to 75.5%, gains of 8.5 and 5.5 percentage points. This comparison measures the aggregate association between the augmentation policy and performance on the fixed test partition. It does not establish that each photometric transformation preserves the maturity information carried by fruit color [39]. Reducing the augmented input from 640×640 to 320×320 decreases mAP50 and mAP50:95 by 2.7 and 2.9 percentage points, respectively, while increasing Tesla T4 throughput from 50.9 to 158.7 FPS, or approximately 3.1-fold. The 320×320 setting is therefore selected as an accuracy–throughput operating point for the matched ablation in Table 5. Table 2 and Table 3 provide the preceding 640×640 architecture-screening context.

### 4.2. Component Ablation and Category-Level Performance

Table 5 separates the roles of BIFA, C2fCIB and SRH through progressive inclusion under the same 320×320 augmented protocol. All GFLOPs values are calculated at 640×640. BIFA is associated with increases of 0.8 percentage points in mAP50 and 1.0 points in mAP50:95 while adding 0.6 GFLOPs. Introducing C2fCIB at the selected neck stage changes mAP50 by a further 0.8 points and reduces complexity by 0.2 GFLOPs, while mAP50:95 changes by −0.1 point. SRH produces the largest computational reduction. GFLOPs decrease from 6.7 to 5.5, or 17.9%, while mAP50 and mAP50:95 change by 0.1 and −0.3 points, respectively. At this step, precision decreases by 3.8 points, and recall increases by 5.0 points, indicating a shift toward retrieving more labeled objects while producing more positive predictions. The GFLOP reduction is deterministic for the specified graph and input size. By contrast, the accuracy changes are single-run point estimates, and differences of 0.1–1.0 percentage points are interpreted descriptively. Functionally, BIFA modifies representation, C2fCIB modifies multi-scale fusion, and SRH simplifies regression for deployment. A dedicated error-diagnosis protocol would be required to further separate localization, class-confusion and background errors [48]. Because the components are introduced progressively, the observed marginal changes are specific to the evaluated sequence. Factorial interactions and run-to-run variability fall outside this design.

Table 6 reports the corresponding class-level values for the same progressive model variants.

Table 6 shows that the largest class-level difference occurs for overripe fruit. Its mAP50 changes from 57.6% to 67.3%, and its mAP50:95 changes from 52.0% to 59.0%. The test partition contains 56 annotated overripe instances, making the 9.7-point mAP50 difference a category-specific single-run estimate based on this sample. The comparatively low YOLO11 baseline identifies overripe fruit as the most difficult class in the evaluated test set. Its color and texture can overlap with ripe, dry or diseased appearances, making retained low-level information and local filtering a plausible explanation for the observed difference. Because the ablation is progressive, the class-specific difference reflects the combined component sequence rather than an isolated BIFA effect. The final model differs from YOLO11 by 0.8 points for diseased fruit, 0.6 points for semi-ripe fruit and 0.5 points for unripe fruit, whereas dry and ripe fruit change by −1.2 and −0.1 points, respectively. These smaller differences are likewise interpreted descriptively under the single-run protocol. Coffee quality guidance and controlled processing studies identify overripe or damaged cherries for separation before subsequent processing [39,49]. The overripe comparison therefore addresses a decision-relevant and comparatively difficult category within this benchmark. Its effect on grading errors or batch quality requires error-specific and process-level evaluation.

### 4.3. Robustness to Visual Degradation and Feature Response

**Figure 9:**
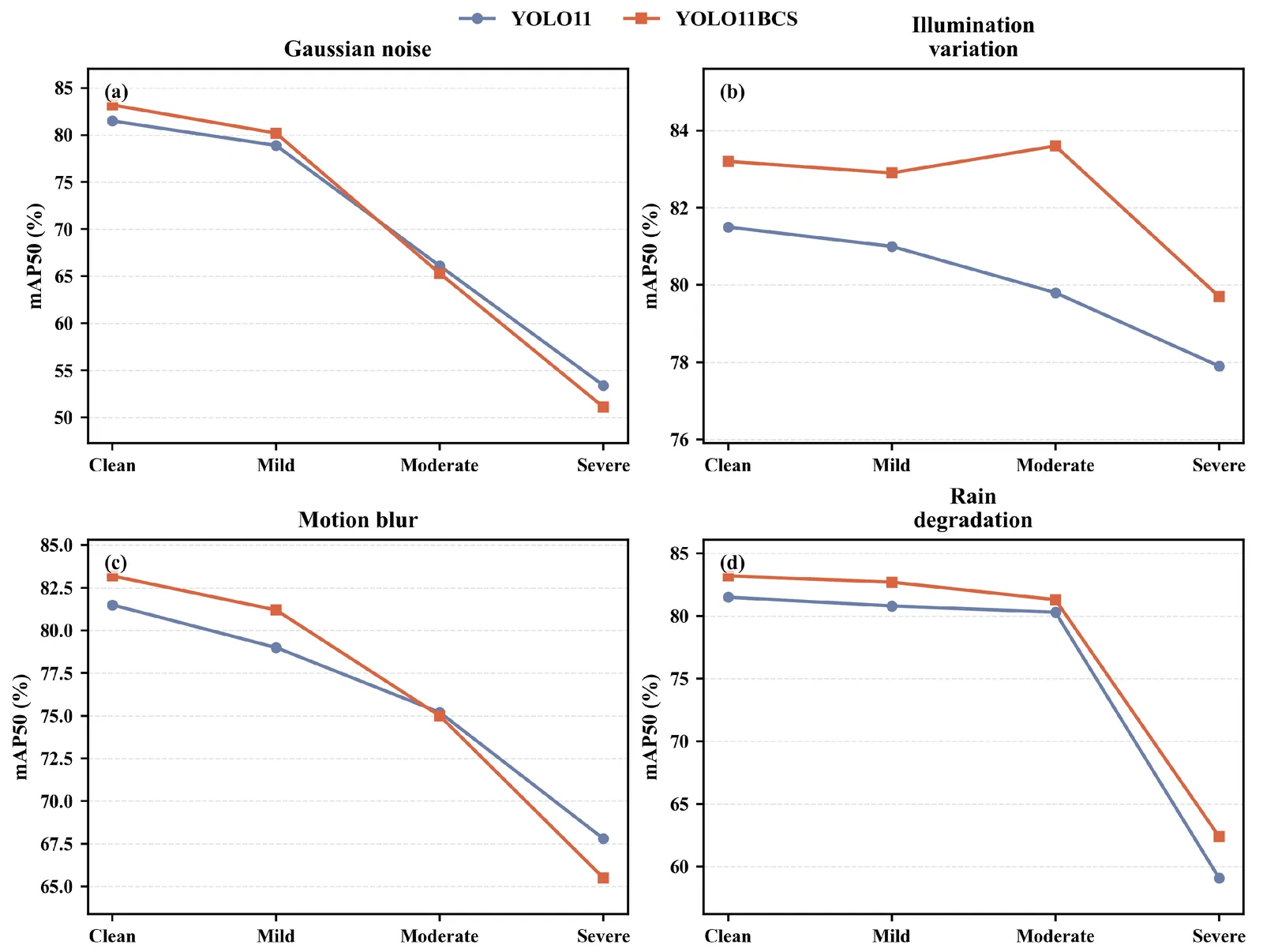
mAP50 trends for YOLO11 and YOLO11BCS under four controlled visual-degradation families.

Figure 9 examines the relative behavior of the two detectors when clean test images undergo controlled visual degradation. Gaussian noise disrupts local high-frequency cues, illumination variation changes global intensity, motion blur removes boundary detail, and rain adds a structured overlay. The complete precision, recall and mAP50 records are provided in Supplementary Table S2. YOLO11BCS differs from YOLO11 by 1.9, 3.8 and 1.8 percentage points under mild, moderate and severe illumination variation, respectively, and by 1.9, 1.0 and 3.3 points under the corresponding rain levels. Within the single-run finite-test setting, differences near one percentage point are interpreted as descriptive observations. The retained path and parallel local transformations in BIFA, together with cross-stage feature reuse, offer a plausible basis for the larger differences under moderate illumination and severe rain. Because each degradation test compares only the baseline and final model, these differences characterize the integrated YOLO11BCS configuration rather than individual BIFA, C2fCIB or SRH contributions. At a moderate illumination reduction, YOLO11BCS reaches 83.6% mAP50, slightly above its clean-set value of 83.2%. This non-monotonic value is consistent with an interaction between the fixed brightness transformation and the finite test set.

The absolute and relative trends define different parts of the robustness profile. Severe Gaussian noise causes the largest decline from the clean condition: mAP50 decreases by 28.1 percentage points for YOLO11 and 32.1 points for YOLO11BCS, reaching 53.4% and 51.1%, respectively. Additive noise perturbs the color and texture cues used to distinguish adjacent ripeness stages throughout the image, which helps explain why local feature aggregation alone does not preserve the advantage at high severity. Severe motion blur removes fruit boundaries and small surface details, producing decreases of 13.7 and 17.7 points and final mAP50 values of 67.8% and 65.5%. By contrast, the spatially coherent rain overlay leaves more low-frequency object structure available, and YOLO11BCS remains 3.3 points higher at the severe rain level. Controlled corruptions provide a repeatable stress test [43], but robustness under synthetic corruptions is not necessarily predictive of robustness to natural distribution shifts [50]. The results therefore establish comparative sensitivity only for the specified transformations. They do not represent joint, spatially varying combinations of rain, motion, illumination and sensor noise in field operation.

**Figure 10:**
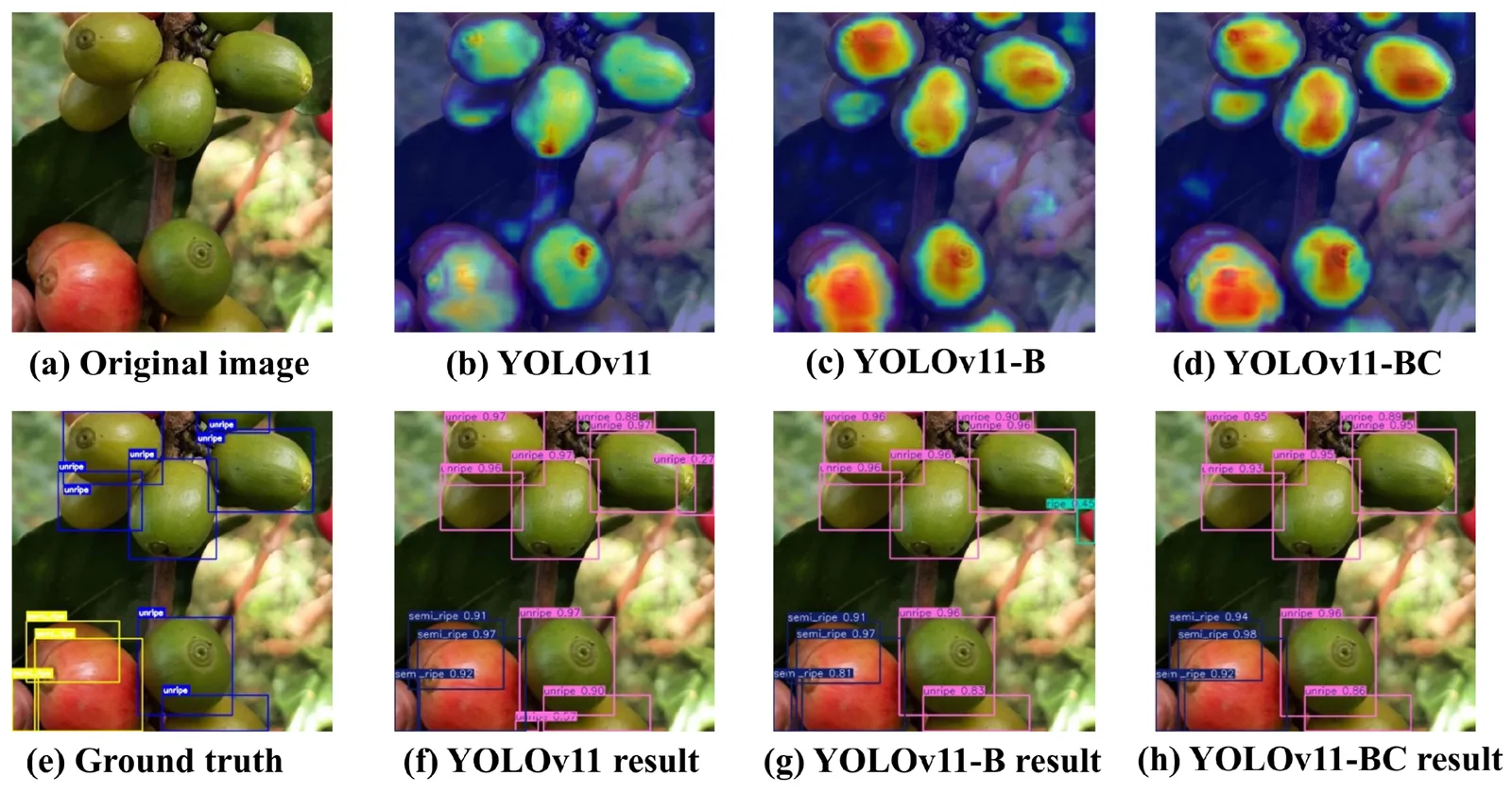
Eigen-CAM responses and detections of YOLO11, YOLO11B and YOLO11BC, showing progressively more concentrated responses over coffee-fruit regions after feature aggregation and fusion.

Eigen-CAM was used to examine spatial responses before the detection head [51]. Figure 10 compares YOLO11, YOLO11B and YOLO11BC at the selected feature layer, which precedes SRH. The final head configuration is therefore outside the scope of this visualization. The displayed cases show more dispersed YOLO11 activation over leaves and background regions, whereas YOLO11B and YOLO11BC concentrate more strongly on coffee-fruit areas. This qualitative pattern is consistent with the mAP50 progression through the feature-aggregation and feature-fusion stages in Table 5. The visualization provides qualitative evidence of feature selectivity rather than a direct estimate of error reduction or an independent module effect.

### 4.4. Embedded Deployment, Energy Efficiency and Cold-Chain Implications

The deployment analysis tests whether the structural differences remain observable after INT8 conversion and whether they extend to throughput and board-level energy use. Before conversion at 320×320, YOLO11 and YOLO11BCS reach 81.5% and 83.2% mAP50, respectively. Their deployed INT8 counterparts reach 81.1% and 82.7%, preserving a 1.6-point difference compared with 1.7 points before conversion. At 640×640, the corresponding INT8 mAP50 values are 83.4% and 85.0%. These values characterize the complete conversion and execution paths, within which quantization and platform-specific effects are combined. Table 7 reports throughput and efficiency. At 640-pixel input, YOLO11BCS increases throughput from 12.8 to 15.9 FPS while both models draw 5.10±0.03 W, raising efficiency from 2.51 to 3.12 FPS/W. At 320-pixel input, throughput increases from 41.2 to 50.3 FPS, while mean power changes from 5.20±0.02 to 5.00±0.04 W, and efficiency increases from 7.92 to 10.06 FPS/W. Runtime memory, model storage and initialization latency affect embedded feasibility alongside arithmetic workload because data movement and processor mapping contribute materially to execution cost [24,25,26]. During continuous inference, the evaluated configuration used approximately 500 MB of runtime memory. This value represents the complete runtime allocation rather than a model-only footprint. Model storage and cold-start latency were outside the measurement boundary.

To express the energy implication in per-image terms, the energy consumed by one processed image is calculated from the board-level power and end-to-end throughput:

(18) $$E_{\mathrm{frame}}=\frac{P_{\mathrm{board}}}{\mathrm{FPS}}=\frac{1}{\mathrm{FPS}/W},$$

where $E_{\mathrm{frame}}$ is the joules per frame, and $P_{\mathrm{board}}$ is the board power in watts. At 640-pixel input, $E_{\mathrm{frame}}$ is 0.398 J/frame for YOLO11 and 0.321 J/frame for YOLO11BCS. The proposed model therefore increases throughput by 24.2% and reduces energy per image by 19.5%. At 320-pixel input, the corresponding values are 0.126 and 0.099 J/frame, so throughput increases by 22.1%, FPS/W increases by 27.0%, and energy per image decreases by 21.2%. The 640-pixel energy difference is associated primarily with the higher completed-frame rate because mean board power is equal, whereas the 320-pixel result combines higher throughput with a 0.20-W lower mean power. The measured quantities include operator mapping, memory movement and host-side stages in addition to nominal GFLOPs. They exclude total operating costs such as equipment, labor, communication and maintenance.

**Figure 11:**
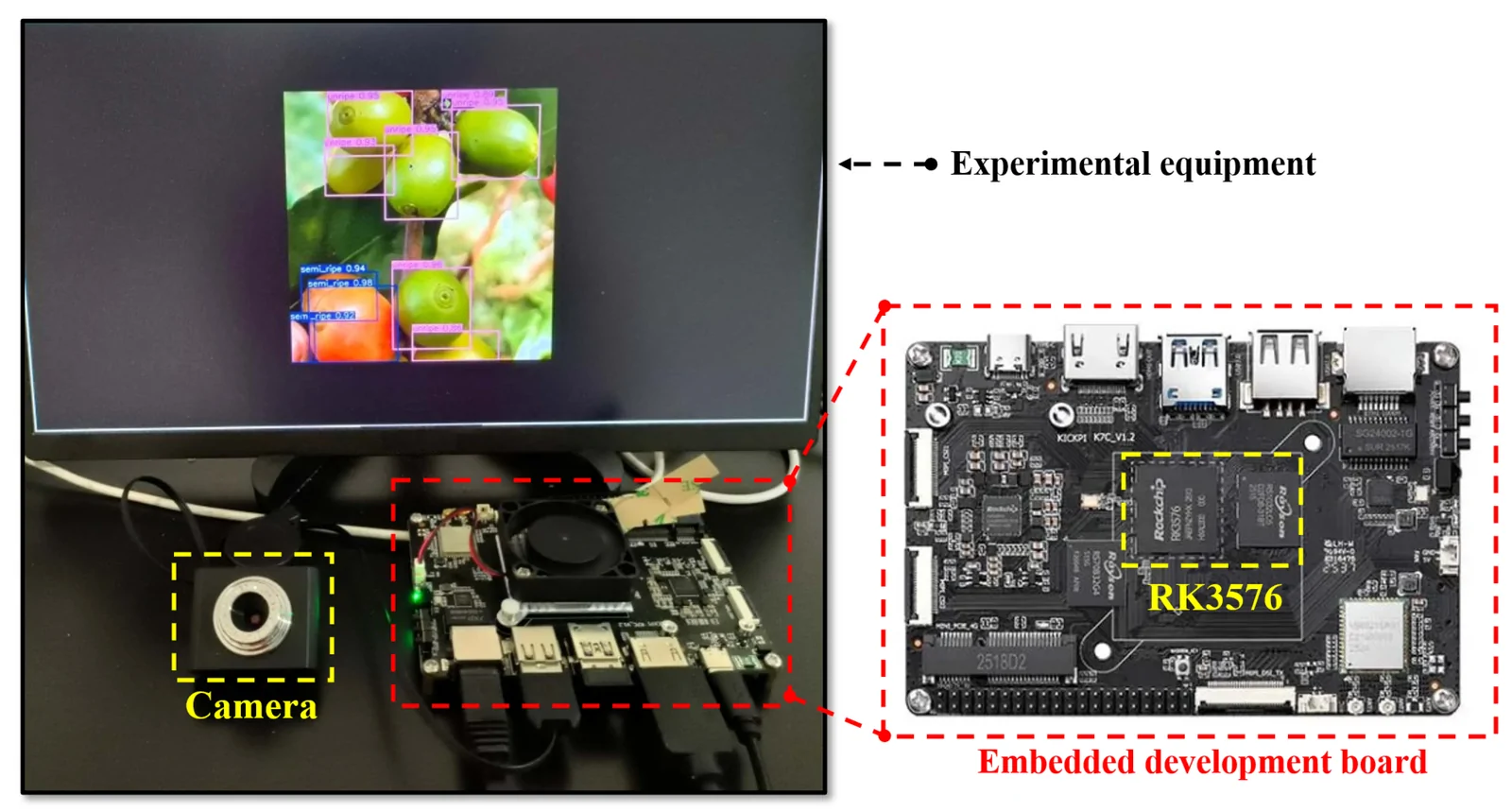
Embedded visual sensing node and RK3576 board-input power measurement arrangement.

Figure 11 shows the physical arrangement used for embedded validation. The camera supplied continuous visual input, while the RK3576 board performed preprocessing, INT8 inference and postprocessing before displaying structured detections. The PA310 connection measured only the board-input power during this continuous execution state.

The reported throughput covers host preprocessing, data transfer, NPU inference and host postprocessing and is therefore broader than network-forward latency alone. In the baseline path, the NPU produces quantized DFL logits and the CPU performs distribution decoding, coordinate reconstruction, candidate filtering and NMS. SRH removes the DFL-specific softmax and expectation operations but retains the same remaining postprocessing settings. C2fCIB also changes dense computation in multi-scale fusion. The RKNN toolchain does not expose a complete operator-level decomposition of latency or memory bandwidth, so the measured FPS and FPS/W represent the combined effect of network structure, quantization and RK3576 execution rather than an isolated estimate of DFL cost. These measurements demonstrate real-time local processing within the stated board boundary. They do not constitute a controlled comparison with cloud inference because network transmission, remote accelerator allocation and server-side queuing were not measured.

From a cold-chain management perspective, class proportions, abnormal-fruit counts and batch timestamps generated by the visual node can complement temperature, humidity and gas records. Visual outputs characterize observed product state, whereas environmental records characterize handling conditions. Their temporal association could support sorting priority, batch isolation, anomaly review and quality traceability, although these operational outcomes were not measured in the present study. At a fixed image volume, the measured 19.5% and 21.2% reductions in board energy per image at 640- and 320-pixel input reduce the execution energy required for local visual processing. Under a fixed board-energy budget, they permit a larger processed image volume. Processing near the data source is commonly motivated by response-time, bandwidth and connectivity constraints [52]. In this system, on-device inference avoids continuous raw-image transmission as a functional requirement and remains available without a network connection. These properties explain the deployment rationale but do not establish superiority over a cloud service, which was not benchmarked. The evaluated system addresses the sensing and visual-inspection layer and does not estimate shelf life, end-to-end logistics cost or facility-level sustainability.

## 5. Conclusions

This study developed a camera-based edge vision system for monitoring product condition near harvest and at the front end of the cold chain. YOLO11BCS combined BIFA for fine-grained feature representation, the YOLOv10-derived C2fCIB configuration for multi-scale fusion and SRH as a reduced regression-channel configuration. Under the matched 320×320 augmented-training protocol, mAP50 changed from 81.5% to 83.2%. At 640×640, computational complexity decreased from 6.3 to 5.5 GFLOPs. The largest category-level difference was 9.7 percentage points for the 56 overripe instances in the test partition. The progressive component sequence showed an accuracy–efficiency trade-off, with single-run accuracy changes that describe the evaluated integration order rather than statistically independent module effects. The clearest deterministic architectural change is the 17.9% GFLOPs reduction associated with replacing the final regression configuration, accompanied by a 0.3-point decrease in mAP50:95 in the evaluated run.

The degradation and deployment results further defined the evaluated operating profile. YOLO11BCS produced higher point estimates than YOLO11 under the tested illumination and rain transformations, with mAP50 differences of 1.8–3.8 and 1.0–3.3 percentage points, respectively. Differences near one point remain descriptive under the single-run design. Under severe Gaussian noise and severe motion blur, YOLO11BCS was 2.3 points below YOLO11. This profile suggests greater tolerance to global intensity changes and structured overlays than to strong corruption of local color, texture and boundaries within the specified synthetic tests. After INT8 conversion at 320-pixel input, YOLO11 and YOLO11BCS achieved 81.1% and 82.7% mAP50, respectively. Throughput increased from 41.2 to 50.3 FPS, and board energy per image decreased by 21.2%. At 640-pixel input, the INT8 models achieved 83.4% and 85.0% mAP50. Throughput increased from 12.8 to 15.9 FPS, and energy per image decreased by 19.5%. Runtime memory use was approximately 500 MB during continuous inference in the evaluated configuration. These measurements support real-time local visual processing within the RK3576 execution boundary, which covers board input but excludes externally powered sensing and communication equipment. A controlled cloud comparison lies outside this measurement boundary.

The evidence covers one public image dataset, a dataset-provided split with 63 training–test source-image correspondences, single-run point estimates, controlled single-degradation tests and validation outside an operating cold-chain facility. The resulting absolute metrics may therefore be optimistic, and the between-model differences characterize this benchmark rather than source-independent field generalization. Transfer across cultivars, regions, seasons and camera systems remains to be evaluated. Further evidence is also needed for run-to-run variability, error-specific diagnostics, combined natural degradations, model storage and startup latency. Hardware-aware compiler scheduling, memory movement and mixed-precision execution remain relevant because embedded energy depends on processor organization and data access in addition to nominal operation count [24,25,26]. Within this scope, the study connects detector design, controlled robustness analysis and board-level energy measurement in one embedded evaluation. Future validation should prioritize source-independent field datasets and naturally co-occurring degradations while retaining direct measurements of compiler behavior and memory movement.

## Figures and Tables

**Table 1:** RKNN quantization and RK3576 deployment configuration.

Setting	Configuration
Conversion path	PyTorch → ONNX → RKNN
Target platform	RK3576 system-on-chip (Rockchip Electronics Co., Ltd., Fuzhou, China)
CPU and GPU	Quad Cortex-A72 + quad Cortex-A53; Arm Mali-G52 MC3
NPU	6 TOPS (INT8); supports INT4, INT8, INT16, FP16, BF16 and TF32
Memory interface	32-bit LPDDR4/LPDDR4X/LPDDR5
Quantization	RKNN hybrid post-training quantization
Calibration data	First 100 images from the training partition
Algorithm and granularity	KL-divergence threshold selection; per-channel quantization
Input and precision	320×320 and 640×640; INT8
Runtime	RKNN-Toolkit-Lite2 NPU inference
Preprocessing and postprocessing	Host CPU

**Table 2:** Architecture screening of candidate YOLO baselines at 640×640 without training augmentation.

Model	P (%) ↑	R (%) ↑	mAP50 (%) ↑	mAP50:95 (%) ↑
YOLOv13n	68.7	79.3	75.6	69.6
YOLOv12n	67.6	77.5	73.0	65.4
YOLO11n	69.5	76.8	75.7	**70.0**
YOLOv8n	70.4	72.8	74.6	69.3
YOLOv5n	72.5	67.7	72.1	67.2

**Table 3:** Descriptive cross-architecture screening at 640×640 without training augmentation.

Model	P (%) ↑	R (%) ↑	mAP50 (%) ↑	mAP50:95 (%) ↑
Faster R-CNN	68.6	77.6	71.4	61.2
RetinaNet	66.7	66.5	66.9	54.6
SSD-MobileNetV3	53.7	51.2	44.7	16.7
RT-DETR	73.9	70.9	70.0	66.0
STN-YOLO	73.6	74.2	75.3	63.8
YOLO11n	69.5	76.8	75.7	70.0

**Table 4:** Effects of training augmentation and input size on YOLO11 performance and Tesla T4 throughput.

Input Size	Training Augmentation	P (%) ↑	R (%) ↑	mAP50 (%) ↑	mAP50:95 (%) ↑	FPS ↑
640	No	69.5	76.8	75.7	70.0	51.1
640	Yes	**84.2**	**82.5**	**84.2**	**75.5**	50.9
320	Yes	77.2	77.9	81.5	72.6	**158.7**

**Table 5:** Progressive ablation of the YOLO11BCS structural components; detection metrics use 320×320 input and GFLOPs are calculated at 640×640.

Model	P (%) ↑	R (%) ↑	mAP50 (%) ↑	mAP50:95 (%) ↑	GFLOPs@640 ↓
YOLO11	77.2	77.9	81.5	72.6	6.3
YOLO11B	80.6	75.7	82.3	**73.6**	6.9
YOLO11BC	79.2	77.1	83.1	73.5	6.7
YOLO11BCS	75.4	**82.1**	**83.2**	73.2	**5.5**

**Table 6:** Clean-test class-level mAP values for the progressive model variants.

Class	Metric	YOLO11	YOLO11B	YOLO11BC	YOLO11BCS
Diseased	mAP50 (%)	91.1	92.6	89.8	91.9
Dry	mAP50 (%)	88.6	86.9	90.3	87.4
Overripe	mAP50 (%)	57.6	63.5	66.3	67.3
Ripe	mAP50 (%)	86.7	87.5	86.0	86.6
Semi-ripe	mAP50 (%)	72.9	70.5	73.1	73.5
Unripe	mAP50 (%)	91.9	93.1	92.4	92.4
Diseased	mAP50:95 (%)	82.5	82.4	80.6	81.7
Dry	mAP50:95 (%)	74.4	72.7	74.5	71.1
Overripe	mAP50:95 (%)	52.0	57.5	59.9	59.0
Ripe	mAP50:95 (%)	78.2	81.4	79.1	79.2
Semi-ripe	mAP50:95 (%)	66.7	64.7	65.4	66.8
Unripe	mAP50:95 (%)	81.6	83.1	81.8	81.4

**Table 7:** Deployment accuracy, throughput and energy efficiency on Tesla T4 and RK3576.

Device	Model	Input	Numeric Format	mAP50 (%) ↑	FPS ↑	Power (W) ↓	FPS/W ↑
Tesla T4	YOLO11	640	FP32	84.2	50.9	32.2 ^†^	1.58 ^†^
RK3576	YOLO11	640	INT8	83.4	12.8	5.10±0.03	2.51
RK3576	YOLO11BCS	640	INT8	85.0	15.9	5.10±0.03	3.12
RK3576	YOLO11	320	INT8	81.1	41.2	5.20±0.02	7.92
RK3576	YOLO11BCS	320	INT8	82.7	**50.3**	5.00±0.04	**10.06**

RK3576 power is reported as mean ± sample standard deviation over three independent 60 s steady-state measurements; FPS/W uses the mean power. ^†^ Tesla T4 power was obtained through NVML, whereas RK3576 power was measured at the board input using the PA310 meter. These values have different measurement boundaries and are not directly comparable.

## Data Availability

The processed dataset, code, trained weights and deployment scripts supporting the findings of this study are available from the corresponding author upon reasonable request.

## Supplementary Materials

The following supporting information can be downloaded at https://www.mdpi.com/article/10.3390/s26206535/s1, Table S1. Principal parameters used to generate the four synthetic degradation families. Table S2. Complete robustness evaluation records for the four degradation families.
