# Supplementary material for: An Energy-Efficient Edge Vision System for Front-End Quality Monitoring in Sustainable Cold Chains: Coffee Fruit Ripeness Detection and Embedded Validation
Source: Sensors (Basel). 2026 Oct 15;26(20):6535. doi: 10.3390/s26206535 (PMC13708895; doi:10.3390/s26206535)
Supplement: Supplementary file 1 [file sensors-26-06535-s001.zip › sensors-4527377-supplementary.pdf]

Supplementary Materials: An Energy-Efficient Edge Vision System for Front-End Quality Monitoring in Sustainable Cold Chains: Coffee Fruit Ripeness Detection and Embedded Validation

Xi Cui [1,2] and Shirong Guo [1,*]

S1. Synthetic degradation generation and parameter settings

Four synthetic degradation families were generated from the original test split to examine representative image-quality changes during outdoor sensing and platform operation: Gaussian noise, illumination variation, motion blur and rain degradation. The corresponding labels were retained without modification, and the generated images were used only for robustness evaluation and visualization. Each degradation family contained mild, moderate and severe levels. The principal severity parameters are listed in Table S1.

Table S1. Principal parameters used to generate the four synthetic degradation families.

Degradation	Parameter	Mild	Moderate	Severe
Gaussian noise	Standard deviation σ	10	20	30
Illumination variation	Brightness factor β	0.7	0.5	0.3
Motion blur	Kernel length l (pixels)	7	13	21
Rain degradation	Mixing coefficient α	0.15	0.22	0.30
Rain degradation	Streak density	0.0010	0.0018	0.0030
Rain degradation	Streak length (pixels)	14	22	30
Rain degradation	Streak width (pixels)	1	1	2
Rain degradation	Blur radius (pixels)	1.1	1.5	2.0
Rain degradation	Brightness factor	0.95	0.90	0.85

Gaussian noise was added independently to the red, green and blue channels as a zero-mean perturbation. Illumination variation was implemented as global brightness scaling. Motion blur used a linear kernel whose direction was sampled within $[-25^\circ, 25^\circ]$ for each image. Rain degradation used a fixed rain intensity of 235 and a streak direction of -18° at all three levels; the density, streak geometry, blur radius and brightness attenuation varied with severity as shown in Table S1. The transformation was applied only to image pixels; the object labels remained unchanged.

S2. Complete robustness evaluation records

Table S2 provides the complete precision (P), recall (R) and mean average precision at an IoU threshold of 0.5 (mAP50) records corresponding to the robustness trends shown in Figure 9. All values were obtained with the 320 × 320 input configuration on the original test split after applying the indicated synthetic degradation. The clean-test mAP50 values used as the starting points in Figure 9 were 81.5% for YOLO11 and 83.2% for YOLO11BCS.

Table S2. Complete robustness evaluation records for the four degradation families.

Degradation	Model	Severity	P ↑ (%)	R ↑ (%)	mAP50 ↑ (%)
Gaussian noise	YOLO11	Mild	77.2	72.5	78.9
		Moderate	63.4	66.4	66.1
		Severe	55.4	59.3	53.4
	YOLO11BCS	Mild	75.4	79.4	80.2
		Moderate	59.2	68.0	65.3
		Severe	55.1	54.1	51.1
Illumination variation	YOLO11	Mild	80.6	73.9	81.0
		Moderate	76.7	77.2	79.8
		Severe	75.5	72.4	77.9
	YOLO11BCS	Mild	76.1	81.2	82.9
		Moderate	81.3	77.3	83.6
		Severe	79.3	72.8	79.7
Motion blur	YOLO11	Mild	75.9	76.7	79.0
		Moderate	70.9	72.6	75.2
		Severe	74.4	59.9	67.8
	YOLO11BCS	Mild	81.1	74.7	81.2
		Moderate	77.4	70.2	75.0
		Severe	72.6	61.8	65.5
Rain degradation	YOLO11	Mild	74.8	79.7	80.8
		Moderate	76.7	78.3	80.3
		Severe	60.4	58.0	59.1
	YOLO11BCS	Mild	76.1	80.8	82.7
		Moderate	74.7	80.6	81.3
		Severe	66.0	57.7	62.4
